# Supplementary material for: Maternal Pre-Pregnancy BMI and Gestational Weight Gain Modified the Association between Prenatal Depressive Symptoms and Toddler’s Emotional and Behavioral Problems: A Prospective Cohort Study
Source: Nutrients. 2022 Dec 30;15(1):181. doi: 10.3390/nu15010181 (PMC9824574; doi:10.3390/nu15010181)
Supplement: Supplementary file 1 [file nutrients-15-00181-s001.zip › nutrients-2141792-supplementary.pdf]

Table S1. The main demographic characteristics of mother-child pairs in different groups

Characteristics	Mother-child pairs included in this analysis n (%) / Mean ± SD	Mother-child pairs not follow-up at 24 months n (%) / Mean ± SD	*p*
Maternal variables			
Age groups, years			0.508
<25	156 (12.83)	186 (12.45)	
25~35	937 (77.06)	1136 (54.77)	
≥35	123 (10.12)	172(11.51)	
Social economic status			0.711
High	550 (45.23)	695 (46.52)	
Low	666 (54.77)	799 (53.48)	
Pre-pregnancy alcohol use			0.968
Yes	394 (32.40)	483 (32.33)	
No	822 (67.60)	1011 (67.67)	
Passive smoking during pregnancy			0.178
Yes	202 (16.61)	220 (14.73)	
No	1014 (83.39)	1274 (85.27)	
Pregnancy complications			0.779
Yes	178 (14.64)	213 (14.26)	
No	1038 (85.36)	1281 (85.74)	
Parity			0.455
Primipara	680 (55.92)	814 (54.48)	

Multipara	536 (44.08)	680 (45.52)	
Delivery mode			0.400
Caesarean	683 (56.17)	815 (54.55)	
Vaginal	533 (43.83)	679 (45.45)	
Pre-pregnancy BMI, kg/m^2	21.54 ± 3.14	21.21 ± 2.87	**0.007**
<18.5	199 (16.37)	251 (16.80)	**<0.001**
18.5~24	737 (60.61)	1014 (67.87)	
≥24.0	280 (23.03)	229 (15.33)	
GWG, kg	13.72 ± 5.48	14.09 ± 5.24	0.166
Inadequate GWG	322 (26.48)	348 (23.29)	**0.036**
Appropriate GWG	511 (42.02)	611 (40.90)	
Excessive GWG	383 (31.50)	535 (35.81)	
Prenatal depressive scores, score	8.70 ± 6.15	8.80 ± 6.23	0.632
Non-depression	600 (49.34)	727 (48.66)	0.935
Sub-threshold depression	470 (38.65)	587 (39.29)	
Depression	146 (12.01)	180 (12.05)	
**Offspring's variables**			
Sex			0.239
Male	634 (52.14)	745 (49.87)	
Female	582 (47.86)	749 (50.13)	
Preterm			0.251
Yes	38 (3.13)	59 (3.95)	

No	1178 (96.88)	1435 (96.05)	
Breastfeeding practice at 6 months old			**0.013**
Exclusive breastfeeding	525 (43.17)	702 (46.99)	
Mixed feeding	407 (33.47)	422 (28.25)	
Artificial feeding	284 (23.36)	370 (24.77)	
